# Using Artificial Intelligence to Achieve Auxiliary Training of Table Tennis Based on Inertial Perception Data

**DOI:** 10.3390/s21196685

**Published:** 2021-10-08

**Authors:** Pu Yanan, Yan Jilong, Zhang Heng

**Affiliations:** School of Computer and Information Science, Southwest University, Chongqing 400700, China; puyanan@email.swu.edu.cn (P.Y.); yanjilong@email.swu.edu.cn (Y.J.)

**Keywords:** wearable computing, inertial sensors, human table tennis action recognition, auxiliary training, fine-grained evaluation

## Abstract

Compared with optical sensors, wearable inertial sensors have many advantages such as low cost, small size, more comprehensive application range, no space restrictions and occlusion, better protection of user privacy, and more suitable for sports applications. This article aims to solve irregular actions that table tennis enthusiasts do not know in actual situations. We use wearable inertial sensors to obtain human table tennis action data of professional table tennis players and non-professional table tennis players, and extract the features from them. Finally, we propose a new method based on multi-dimensional feature fusion convolutional neural network and fine-grained evaluation of human table tennis actions. Realize ping-pong action recognition and evaluation, and then achieve the purpose of auxiliary training. The experimental results prove that our proposed multi-dimensional feature fusion convolutional neural network has an average recognition rate that is 0.17 and 0.16 higher than that of CNN and Inception-CNN on the nine-axis non-professional test set, which proves that we can better distinguish different human table tennis actions and have a more robust generalization performance. Therefore, on this basis, we have better realized the enthusiast of table tennis the purpose of the action for auxiliary training.

## 1. Introduction

As China’s “national ball”, table tennis was introduced to China in 1904. After a century of precipitation and development, it has been deeply loved by most Chinese people. Today, China’s table tennis development trend is unstoppable, and China’s table tennis is at the top international level in terms of echelon construction and talent allocation [1]. Since the COVID-19 outbreak at the end of 2019, the rise of national fitness has become China’s national strategy, and table tennis, as China’s “national ball”, has become the sport of choice for people of all ages in China. Moreover, the international community also has many enthusiasts. However, table tennis has high technical content, professionalism, and practicality. In the actual sports process, for amateur athletes, if they cannot grasp the key points of the relevant batting actions and footwork, or in the course of sports, wrong actions and non-compliance with the rules and requirements of the action may result in injuries such as ligament injury or muscle strain [2]; especially for some more challenging actions, such as the Loop and Speedo, violating the fundamental laws of the body and the principles of sports mechanics is more likely to cause different degrees of injury to athletes.

The general public does not have professionals to guide their action in table tennis. Blind exercise can easily cause damage to their own body. With the development of technology, the guidance of table tennis actions does not necessarily require professionals. Information and computer auxiliary training has become a hot spot. Chu et al. [3] designed a running assistant training device based on radio frequency technology. Jiang et al. [4] analyzed the application of cloud computing technology in sports-assisted training. Zhou et al. [5] applied embedded systems and virtual reality to sports training and pointed out the application of virtual reality in physical education.

At present, the definition of human action is relatively broad. According to the semantic level of human behavior, Moeslund et al. [6] divided human actions into the following three levels: Action Primitive, Action, and Activity. Action atoms are the basic units that make up human actions; actions are repetitive whole-body movements composed of action atoms; activities include a series of actions. According to the complexity of human behavior, Vishwakarma et al. [7] divided human behavior into the following four levels: Gesture, Action, Interaction, and Group Activity. Secondly, the definition of human action recognition is the recognition of interaction between people, between people and things, and between people and the environment. According to the complexity and semantic level of human actions, Zhang et al. [8] can classify human action recognition into the following four categories: original action recognition [9], single-person action recognition [10], interactive recognition [11], and group action recognition [12]. According to the different types of data used for human action recognition, human action recognition is usually divided into the following three main categories: human action recognition based on vision, human action recognition based on acoustics, and human action recognition based on inertial sensors [13]. Visual action recognition extracts human action data from image or video data obtained by optical sensors [14,15], acoustic action recognition uses sound signals for high-precision hand action tracking and gesture recognition [16,17], inertial sensor action recognition focuses on extracting human action inertial data from wearable inertial sensors [18,19]. Liu Yutao [20] summarized the three methods, as shown in the following Table 1.

Human action recognition based on vision and acoustics is constantly evolving and is superior to human action recognition based on inertia in some aspects. However, its recognition performance is still limited by many objective conditions, such as complex backgrounds, lighting conditions, camera angle shifts, changes in human and object signs (height, weight), etc., which will interfere with visual action recognition. Environmental noise and small detection areas also bring inconvenience to acoustic action recognition [21]. At the same time, the privacy and ethical issues brought about by cameras and microphones will also cause people to worry [22,23]. Human action recognition, especially in sports, often requires conditions such as complex stage scenes, high-speed displacement, and multiple people on the same stage, which is not conducive to applying human action recognition based on vision and acoustics. To cope with these shortcomings and challenges, many researchers have proposed using wearable inertial sensors to realize human action recognition [24,25].

The human body action recognition based on wearable inertial sensing has the advantages of minor space limitation, little external environment interference, simple device use, and user privacy protection. At the same time, wearable inertial sensing can capture local human action data, which is convenient for fine-grained evaluation of local human action. We research human ping-pong action recognition and evaluation based on wearable inertial sensors, which are used to assist in ping-pong action training, help people improve ping-pong action skills, and promote people to enhance human health through ping-pong exercise. Our main contributions are as follows: (1) Proposed window segmentation point detection and keyframe extraction methods. (2) The MDFF-CNN model is proposed to improve the accuracy and generalization of human action recognition. (3) The overall and fine-grained evaluation methods of ping-pong action are proposed. In addition, because the proposed solution is based on action recognition, we explain the human table tennis action recognition and human table tennis action evaluation in the human action recognition process. Critical process and innovation. In the rest of this article, the following structure will be followed:

- Section 2 introduces our crucial preparation work.
- Section 3 presents the two critical processes of human table tennis action recognition and human table tennis action evaluation in detail.
- Section 4 provides the results to verify the performance of the method.
- Section 5 summarizes the significant developments and their impact and provides ideas for future work.

## 2. Preparation Work

### 2.1. Coordinate System Definition and Inertial Sensors

**Figure 1:**
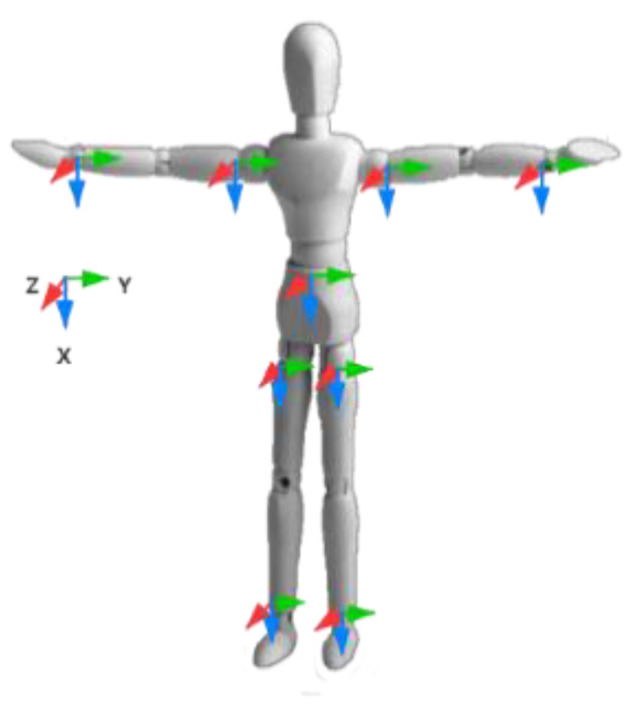
Human body and sensor coordinate system based on bone nodes.

To describe the ping-pong action, it is necessary to construct a coordinate system based on the human body’s activity. In the wearable human body action capture system, the following three coordinate systems are mainly involved: the reference coordinate system, the sensor coordinate system, and the human body coordinate system. To ensure the accuracy of the experiment, all our investigations are carried out in the same coordinate system. We establish a human body coordinate system for each bone node of the human body. The human body coordinate system will change in real-time with the movement of the human body joints. It can represent an absolute change relative to the reference coordinate system, or it can be expressed as a relative change relative to the human bone node. We construct a human body coordinate system for the human bone nodes. The positive direction of the *X*-axis points to the human body downwards, the positive direction of the *Y*-axis points to the left of the human body, and the positive direction of the *Z*-axis is perpendicular to the human body forward in Figure 1.

**Figure 2:**
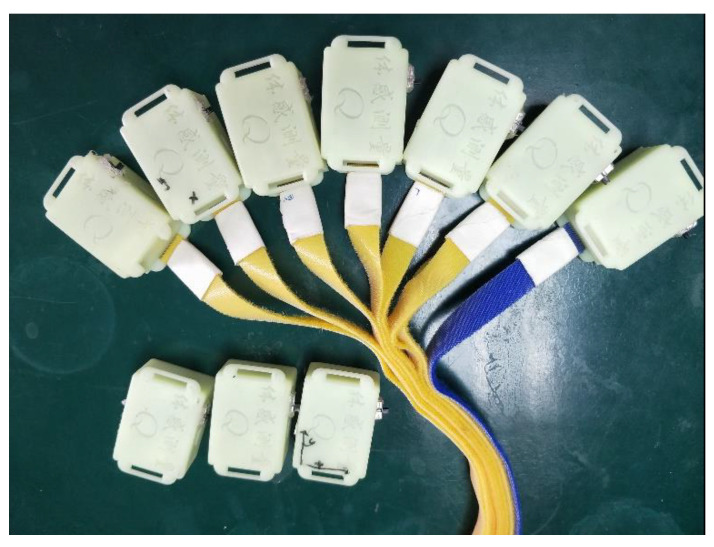
Hang3.0 inertial sensors schematic diagram.

We chose the third-generation inertial sensor (Hang3.0), made by ourselves, as the experimental sensor, as shown in Figure 2. Hang3.0 can accurately judge the standing posture, squatting posture, lying posture, walking posture, and running posture of the human body. The accuracy of human posture judgment: >95%; the static accuracy of human posture roll/pitch: <0.03° RMS; Human body attitude roll/pitch dynamic accuracy: <5° RMS; maximum data acquisition frequency is 50 Hz; attitude angle resolution is 2°; compared to the popular Perception Neuron PRO (45800RMB) and FOHEART·X (15800RMB) Sensor, our sensor only cost 1000RMB in production costs. Although the indicators of our sensors are not as good as those of the two sets of devices, it also proves that the method we propose still has a specific effect in the field of additional ping-pong training under extreme environments.

### 2.2. Human Body Action Representation Based on Inertial Data

In this article, we wear multiple wearable inertial sensors on different joints of the human body, that is, many-to-many wearable inertial human action recognition settings, and use the sensed human action inertial data to formally describe human motions.

Each inertial node senses the acceleration, a, angular velocity, w, and magnetic field information, h, at a time, t, through the accelerometer, gyroscope, and magnetometer sensor inertial data on the three axes of *x*, *y*, and *z*. The length of the three-axis magnetic field is nine one-dimensional vector *N*, which is expressed as follows:

(1) $$N\left(t\right)=\left[a_{x}\left(t\right),a_{y}\left(t\right),a_{z}\left(t\right),w_{x}\left(t\right),w_{y}\left(t\right),w_{z}\left(t\right),h_{x}\left(t\right),h_{y}\left(t\right),h_{z}\left(t\right)\right]$$

Assuming that there are d sensors worn on different joint points of the human body, the one-dimensional observation vector, S, composed of the sensor node’s perception data at time  t can be expressed as follows:

(2) $$S\left(t\right)=\left[N_{1}\left(t\right),N_{2}\left(t\right),\cdot \cdot \cdot ,N_{d}\left(t\right)\right]$$

Assuming that the observation time of each action is T, the two-dimensional observation matrix of an action, M, formed by the perception data of d sensors within T can be expressed as follows:

(3) M=[[S(1)],[S(2)],···,[S(t)],···,[S(T)]]

The number of rows of the action matrix M is T, and the number of columns is 9×d.

Assuming that there are i actions in each category, the action data set *A* of this category can be expressed as follows:

(4) $$A=\left\{M_{1},M_{2},\cdot \cdot \cdot ,M_{i}\right\}$$

Assuming that our action data set has a total of j action categories, the complete action data set $A^{*}$ can be expressed as follows:

(5) $$A^{*}=\left\{A_{1},A_{2},\cdot \cdot \cdot ,A_{j}\right\}$$

### 2.3. Wearable Inertial Sensing Data Window Segmentation Point Detection and Key Frame Extraction

At present, the segmentation of data signals can be mainly divided into three categories, namely, sliding window segmentation, event window segmentation, and active window segmentation. Sliding window segmentation has the advantages of simple method, fast speed, and sound effect. However, a smaller window is likely to cause a poor recognition effect, and a larger window is expected to contain multiple data segments, resulting in erroneous data. There is currently no clear consensus on which window size should be used. Event window segmentation can accurately segment the motion signal by recording the start and end time of the action. However, because it requires additional measurement equipment or related algorithms to determine the beginning and end of the window during use, the application of this method is not shared. Active window segmentation needs to set related algorithms for segmentation according to the data signal characteristics of different actions. However, the judgment of the various attributes between other action signal data will affect segmentation accuracy.

Banos et al. [26] evaluated some of the most widely used action recognition processes for various window sizes. The results showed that the best recognition could be obtained by keeping the window interval length at 1–2 s. Wang et al. [27] compared the effects of different window sizes on human action and gesture recognition based on smartphone sensors. The results confirmed that the window length has a significant impact on motion pattern recognition. The window length of 2.5–3.5 s can provide the best motion recognition results. Aminian et al. [28] proposed a new method that uses only two miniature accelerometers and a portable digital recorder to detect the gait cycle phase and analyze the gait from the accelerometer signal. Selles et al. [29] installed two single-axis accelerometers under the subject’s knees. They recorded the reaction moment of the ground as the start and end of the walking action to obtain the accelerometer window data. Nyan et al. [30] use wavelet analysis to separate different action windows, and each window represents a complete human action. Yan et al. [31] designed a complex behavior boundary discrimination algorithm based on multiple windows to distinguish numerous complex actions.

To select the appropriate data signal segmentation method, we analyzed the law of table tennis movement. We found that the right wrist of the right-handed athlete has strong explosive power when hitting the ball, and the hand posture changes most obviously. Wearing an inertia node on the wrist to perceive the change of table tennis action makes it easier to judge the beginning and end of table tennis action by analyzing inertial data. Taking the real-time data of human table tennis movement sensed by the inertial sensor worn on the hand as the research object, the hitting action is divided into “two processes, three moments”, namely, two processes of hitting and resetting, and three of initial, hitting, and termination moments. The process of table tennis hitting action is from the beginning, the hitting process, the acceleration gradually increases; the hand reaches the farthest point, the acceleration slowly reaches the highest point, and hits the table tennis ball in the process (for convenient analysis, we assume that the hand reaches the farthest point at the hitting moment); after that, the writing begins to reset, and the acceleration gradually decreases; finally, when the hand reaches the end position, the acceleration returns to a static state. We assume that the inertial data in the table tennis action window obey a normal distribution.

Furthermore, acceleration represents the change of the linear velocity and direction of the rigid body concerning time and is not affected by the transformation of the magnetic field. Using the three-axis synthetic acceleration window for analysis can show the process of table tennis action more clearly.

**Figure 3:**
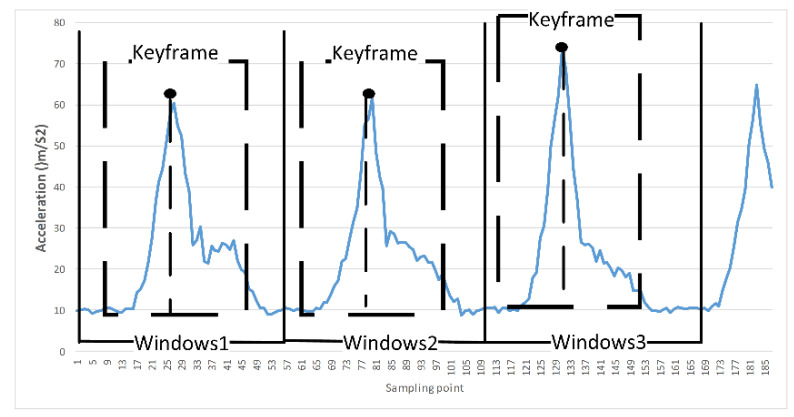
Schematic diagram of human table tennis action data window and key frame.

Then, the “3σ” principle of the normal distribution is extended to the inertial window data. A small part of the data on the left and right sides of the hitting moment contains most of the action characteristics. The table tennis ball hitting action generally lasts 1 to 2 s, and the data are collected at a rate of 30 frames per second, and the action window data of about 30 to 60 frames can be obtained. Therefore, for the subsequent modeling and calculation, we define the window data of 36 frames in a total of 18 structures on the left and right sides of the hitting moment as action keyframes according to the “3σ” principle of normal distribution, as shown in Figure 3. It is expected that fewer data can be used to represent the action window data, and the data frames whose actions are not very representative are removed, while ensuring that the extracted keyframes can completely describe the real action.

**Figure 4:**
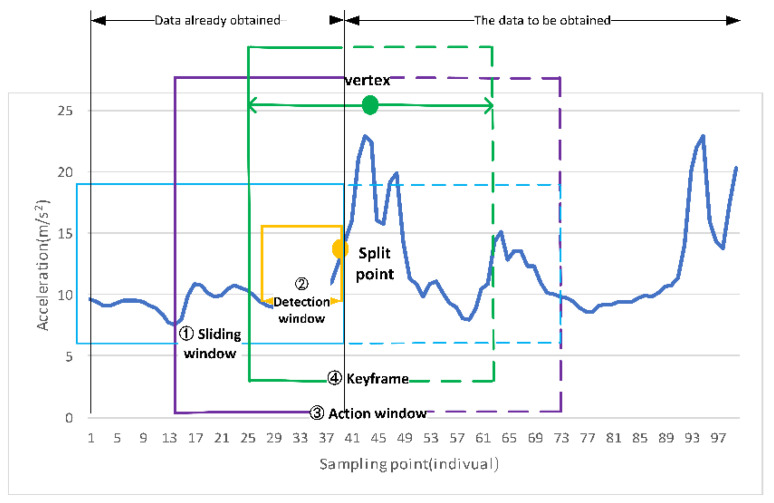
Schematic diagram of window segmentation point detection and key frame extraction method based on human table tennis action data.

To extract keyframes from the synchronized real-time inertial data, we propose window segmentation point detection and keyframe extraction methods, as shown in Figure 4.

Use a queue with a length of L as the first-layer window to store the received inertial data frame, which is called a sliding window. When the maximum length L is reached, the first-in, first-out strategy is used. In the sliding window of the first layer, a small data frame at the end of the queue is used as the second-layer window, called the detection window. The main function is to analyze the data signal changes in the window to determine whether the user has hit the ball. Additionally, define the starting position of the hitting action as the window split point. From the window split point, a fixed-length queue window is generated to save the user’s action sequence in the next period of time, and this window is called the action window. According to the “3σ” principle of a normal distribution from the action window, 36 frames of action keyframes are obtained from the action window.

## 3. Implementation of Key Models and Methods

Our primary research content is a wearable inertial perception ping-pong action-assisted training method. It mainly solves the two critical processes of human ping-pong action recognition and human ping-pong action evaluation in the human body action recognition process. Based on this, a method of assisted training of human ping-pong movement is proposed.

### 3.1. Multi-Dimensional Feature Fusion Convolutional Neural Network

While acquiring the keyframes of the table tennis action, some of the action data are also lost, which brings challenges to the subsequent action recognition effect. Although the research on human action recognition using artificially extracted features has achieved objective results, there are still many shortcomings. In contrast, deep learning methods have strong autonomous learning capabilities, can automatically extract features from training data, do not require manual feature design, and have high recognition accuracy.

To improve the effect of the neural network, the most direct method is to expand the network structure, including increasing the depth and width of the network. However, this simple network structure brings the following two main disadvantages: first, the extended network structure means more weight parameters, which makes the expanded network more prone to overfitting, especially when the number of training sets is limited; Second, increasing the network size will sharply increase the amount of calculation. If the two convolution layers are a chain, the increase in filters may aggravate the amount of analysis, resulting in a shortage of computing resources and an inability to complete the network training quickly.

The key to solving these two problems is to transform the fully connected architecture into a sparsely connected architecture, even in the interior of the volume layer. Szegedy et al. [32] proposed a deep convolution neural network architecture code-named “inception”. The main feature of this structure is to improve the utilization of computing resources in the network, increase the depth and width of the network, reduce network parameters and reduce the amount of calculation.

**Figure 5:**
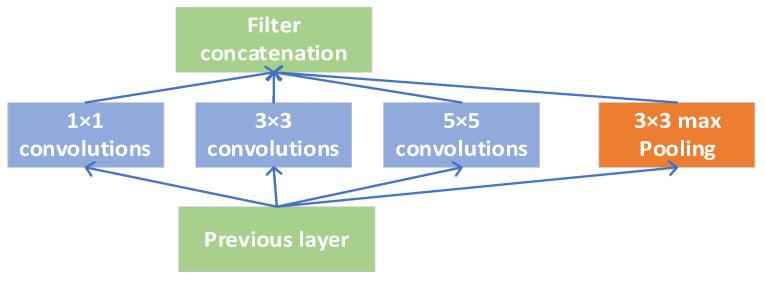
Naive Inception module.

Figure 5 shows a simple caption module, which connects 1 × 1, 3 × 3, 5 × 5 convolution, and 3 × 3 Max pooling together to obtain nonlinear attributes, form an output vector, and input to the next level. Each layer in the network can learn the characteristics of “sparse” (3 × 3, 5 × 5) or “not sparse” (1 × 1). On the one hand, this processing increases the width of the network; on the other hand, it increases the adaptability of the network to multi-scale filtering. The concept module also includes a maximum pool. The purpose of this is to increase the adaptability of the network to different scales and strengthen the recognition ability of the convolutional neural network to various size features.

**Figure 6:**
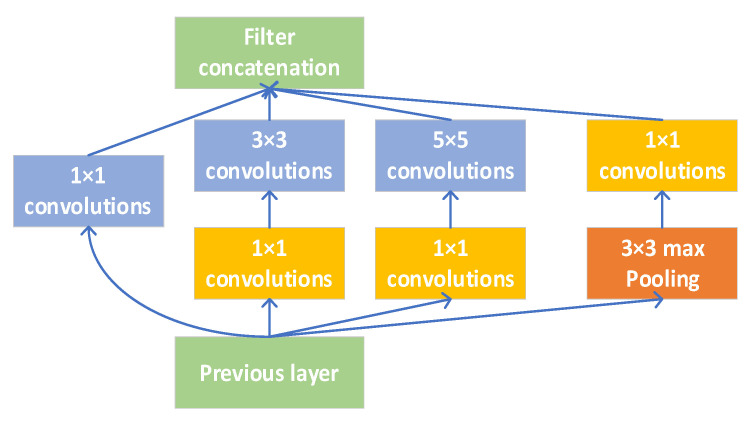
Inception module with dimensionality reduction.

However, under this simple Inception model, the feature map after the output is connected in series is still huge, although the performance can be improved. After passing through the multi-layer Inception network, it will cause too many parameters and face the problem of a large amount of calculation. To solve this problem, the Inception module shown in Figure 6 is used.

Compared with the simple Inception module, in the Inception module with dimension reduction, a 1 × 1 convolution layer is added in front of a 3 × 3 convolution layer, and a 5 × 5 convolution layer, respectively, and a 1 × 1 convolution layer is added after the maximum pool layer. The input of the previous layer is reduced through the 1 × 1 convolution layer, which allows the number of neurons to be significantly increased in each stage without causing an uncontrolled expansion of computational complexity.

**Figure 7:**
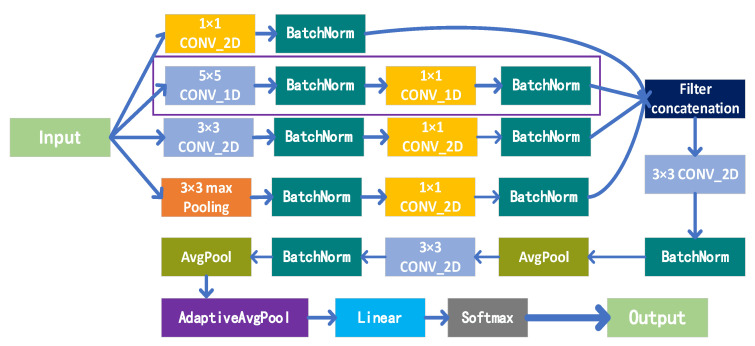
Schematic diagram of the overall architecture of the MDFF-CNN model.

We use the advantages of the Inception network structure to expand the network width and depth, which can significantly reduce the weight parameters in the network and reduce the computational complexity, and improve the utilization of computing resources within the network. An improved Inception network structure is proposed. In the Inception network structure, one-dimensional and two-dimensional spatial feature maps are obtained through 1D and 2D convolution kernels, and then the cascaded feature maps are input into the convolutional neural network for human action recognition research. The network is named Multi-dimensional Feature Fusion Convolutional Neural Network (MDFF-CNN). The architecture of our proposed MDFF-CNN model is shown in Figure 7.

The MDFF-CNN proposed by us has the following advantages:

(1) The introduction of a 1 × 1 convolution kernel is used to organize information across channels and reduce the dimensionality of input channels, which effectively improves the expressive ability of the network. Using the cross-channel capability of the 1 × 1 convolution kernel, the features that are highly correlated but are in different channels at the same spatial location are connected together. In addition, the 1 × 1 convolution kernel has a small amount of calculation, and it can also add a layer of features and nonlinear changes.
(2) In order to avoid the excessive number of convolution maps after cascading, we adjusted the 1 × 1 convolution to the 3 × 3 convolution in the improved Inception network and reduced the dimensionality of the output feature map.
(3) The global average pooling (AdaptiveAvgPool) method is used to replace the fully connected layer. The fully connected layer occupies most of the parameters in the convolutional neural network, which is the most prone to overfitting and the most time-consuming place in the entire network. The global average pooling method has the following advantages: First, it can better associate the category with the feature map of the last convolutional layer. The second is to reduce the number of parameters. The global average pooling layer has no parameters, which can prevent overfitting in this layer. The third is to integrate global spatial information. For the convenience of classification, we still use the fully connected layer in the last layer of the network. However, after the global pooling process, the amount of data entering the fully connected layer has been greatly reduced.
(4) We have combined the advantages of multiple network structure designs and methods, which can effectively reduce network complexity and weight parameters. It is expected that a better recognition speed can be obtained on the premise of maintaining the recognition effect.

### 3.2. Human Table Tennis Action Assessment Method

We prove, through experiments (see Section 4 for the experimental part), that the multi-dimensional features learned through the MDFF-CNN model have a stronger representation ability for table tennis actions. On this basis, we design a human table tennis action assessment method. Human table tennis action assessment includes an overall assessment of human table tennis action and a fine-grained assessment of human table tennis action. The overall assessment of human table tennis action is to analyze the similarity between human table tennis action and standard action, and the fine-grained assessment of human table tennis action is to analyze the difference between human body local table tennis action and local standard action. Both can use similarity to express action evaluation results.

#### 3.2.1. Overall Assessment of Human Ping-Pong Actions

Usually, the Softmax function is added to the last layer of the neural network to turn the output of the neural network into a probability distribution output. The output of the probability distribution can be understood as the derivation of the neural network, the probability that a sample is divided into different categories, and the category label corresponding to the largest probability value is used as the classification result of the sample. We take this maximum probability value as the evaluation of the similarity between the overall human table tennis movement and the standard movement, which has a certain reference value.

The Softmax function expression is shown in Formula (6) as follows:

(6) $$y_{i}=Softmax\left(x_{i}\right)=\frac{e^{x_{i}}}{\sum_{i=1}^{n}e^{x_{i}}}$$

#### 3.2.2. Fine-Grained Evaluation of Human Table Tennis Actions

**Figure 8:**
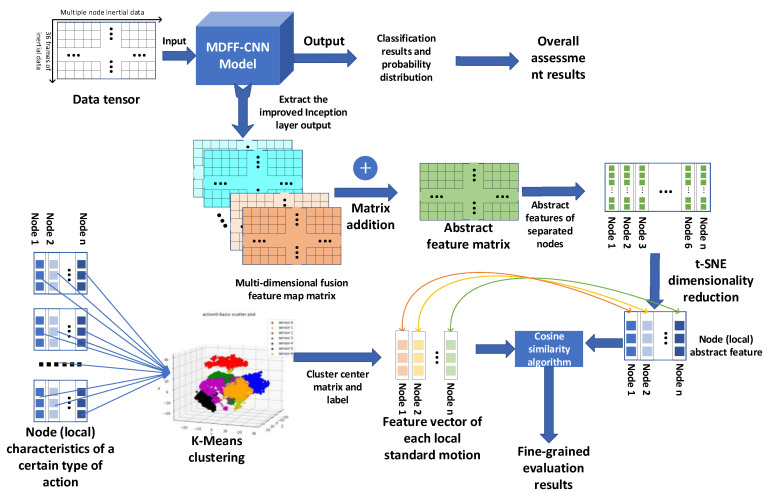
Schematic diagram of table tennis action evaluation algorithm.

We propose a fine-grained evaluation algorithm for human table tennis action, as shown in Figure 8. The algorithm is divided into the following two processes: The first is to construct a standard model of human table tennis local action. The second is to calculate the similarity between the local human body movement and the standard model of the human body movement at that position.

The primary task of a fine-grained assessment of human table tennis action is to construct a standard human table tennis action model for each node of the human body. Taking the Forehand Stroke as an example, we construct the standard action feature vector of each partial action of the Forehand Stroke, and the standard action feature vectors of all the parts that constitute the standard action model of the Forehand Stroke.

First, input the inertial data (tensor) of each Forehand Stroke in the human table tennis exercise training set into the trained MDFF-CNN network. The one-dimensional and two-dimensional features of the inertial data can be obtained through the output of the improved Inception network structure picture. Since the improved Inception network structure only performs multi-dimensional feature extraction and does not change the shape and structure of the input tensor, the multi-dimensional feature map after the improved Inception processing can be regarded as the high-level abstract feature of the input data. The data have a certain characterization significance. The matrix addition operation is performed on the output multi-dimensional fusion feature map, and all the feature maps are merged to obtain the abstract feature map of the input action.

Then, according to the combined order of the inertial nodes, the fused abstract feature maps are separated to obtain the abstract features of different inertial nodes. By operating all the Forehand Strokes in the training set according to this method, an abstract feature data set of all the Forehand Strokes can be obtained. The t-SNE algorithm is used for dimensionality reduction, and the three-dimensional feature vector of each local feature is obtained. t-SNE has the same characteristics of the separability of the original data and the low-dimensional spatial data. We use t-SNE to reduce the dimensionality of local action abstract features. The purpose of this is to speed up the K-means clustering algorithm and similarity calculation. At the same time, we can use visualization to determine whether the features belonging to different nodes are in a low-dimensional space.

After that, all the local feature data sets of the Forehand Stroke are classified by the K-means clustering algorithm. K-means algorithm is a clustering algorithm based on partition. It uses K as a parameter to divide n data objects into K clusters (categories) so that the clusters have a higher degree of similarity, while the similarity between clusters is relatively high. We use the K-means clustering algorithm to set the number of clusters, K, to the number of nodes worn. Then, the cluster center of each category after clustering is regarded as the “representative” action feature of the category; that is, the cluster center data represent the standard action feature of the local location. Since the K-means algorithm is an unsupervised algorithm, we do not know the true label (node number) corresponding to each type of cluster center. We can match each learned cluster label with the true label to obtain the truth of the cluster center. The label is the true category of the cluster center.

## 4. Experiment and Result Analysis

### 4.1. Data

We selected 12 professional table tennis players as subjects. Each subject wore inertial sensors as required and then continuously made the same type of table tennis shots within a certain period of time and collects inertial data. The personal attributes of these 12 athletes are shown in Table 2:

It can be seen from the data in the table that our subjects included male and female genders, with a height range of 155~185 cm and a weight range of 47~80 kg. Due to the different attributes of the subjects, the collection of actions of the same category from different subjects has certain intra-class differences.

**Figure 9:**
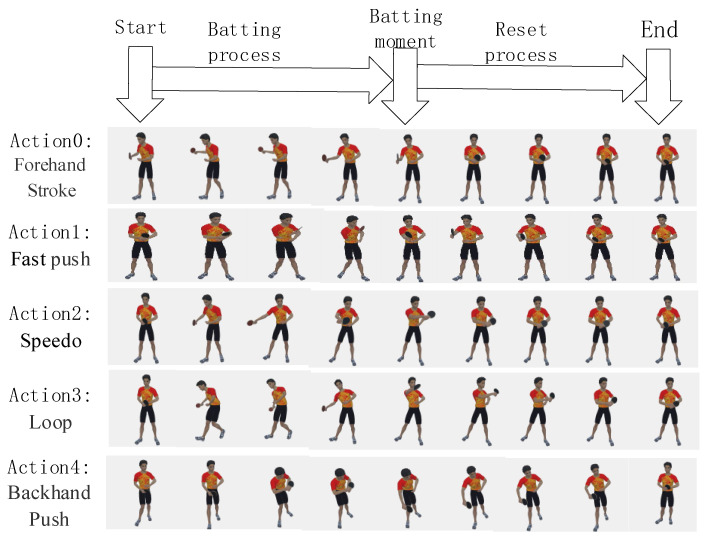
A simple schematic diagram of the five-table tennis hitting processes.

We collected the inertial data of five common table tennis ball hitting actions. The five table-tennis hitting actions are Forehand Stroke (action 0), Fast push (action 1), Speedo (action 2), Loop (action 3), and Backhand Push (action 4); these five actions are the basic actions of table tennis, considering the complexity and similarity of table tennis. We asked each participant to perform the five actions several times in turn, and collected 200 table tennis moves of each category, a total of 1000 table tennis shots, and a total of 62,803 frames of data for experimentation. The collected five kinds of table tennis hitting action data are shown in Table 3, and the five kinds of table tennis hitting processes are shown in Figure 9.

We used the data of person 0 and person 1 as major-test sets. After mixing the data of the remaining ten subjects, they were randomly divided into training sets and validation sets. The data distribution is shown in Table 4.

In addition, in order to further verify the classification effect and generalization ability of the classification model for table tennis action, we additionally took eight non-professionals as subjects and collected 180 table tennis action data of each category according to the same method for a total of 900 data as a non-professional test set (amateur-test sets). These eight non-professionals also have the attributes of men and women, tall and short, fat and thin. They have almost never been exposed to table tennis. The data collected is to follow professional athletes to make simulated table tennis actions. The reasons for collecting the non-professional test set are as follows: First, the non-professional test set has more obvious inter-class similarity and intra-class difference, and the recognition effect of the classifier in the non-professional test set can better explain the classification and generalization of the classification model’s performance. Second, our goal is to identify action types for users and conduct action assessments, assist them in improving table tennis action training in a targeted manner so as to help people improve their table tennis skills and enhance human health through exercise. Therefore, the classification model has more guidance and application value in a non-professional test set.

### 4.2. Multi-Dimensional Feature Fusion Convolutional Neural Network Experimental Results

We used the following two model evaluation methods to evaluate the model: F1-Score and confusion matrix. The accuracy of model recognition is reflected by the F1-Score while taking into account the accuracy and recall of the classification model. Generally speaking, the actual accuracy displayed will be extra due to the different calculation methods of the F1-Score and the confusion matrix.

#### 4.2.1. Traditional Machine Learning Action Recognition Experiment and Result Analysis Based on Artificial Features

Before the formal experiment, to be more convincing, we first experimented with traditional machine learning action recognition methods based on artificial features. We used traditional machine learning models for data modeling. The classification models included KNN, SVM, Gaussian Bayes (GaussinNB, GNB), and Random Forest (RandomForest, RF), four traditional classifiers. First, we regularized each keyframe data window in the data set to eliminate the influence of different dimensions. Then, feature extraction was performed on each keyframe data window, and the time domain feature and frequency domain feature of the action keyframe data were extracted.

Then, we used PCA to reduce the dimensionality and set the variance of the principal components to 0.9 to obtain the reduced feature vector, and divided it into the training set, test set, and validation set at a ratio of 7:3. Finally, the training set after feature extraction was used for model training, and the test set was used to test the model classification effect.

(1) The recognition result of the classifier on the professional test set

**Figure 10:**
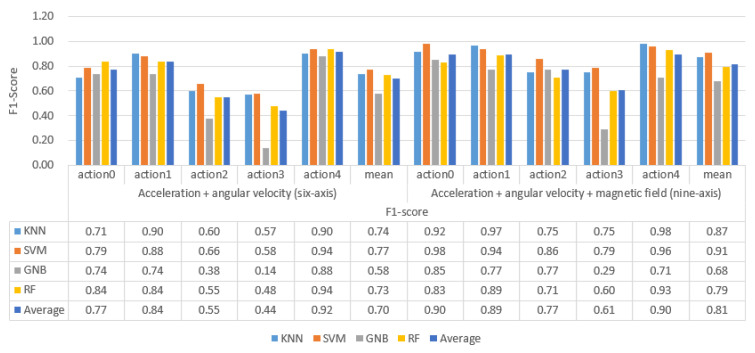
The recognition accuracy of traditional machine learning models based on artificial features on professional test sets.

We used 6-axis and 9-axis training data sets to train the four classifiers. Then, the trained classifier was used to identify and classify the professional test set, and the recognition accuracy F1 value is shown in Figure 10.

**Figure 11:**
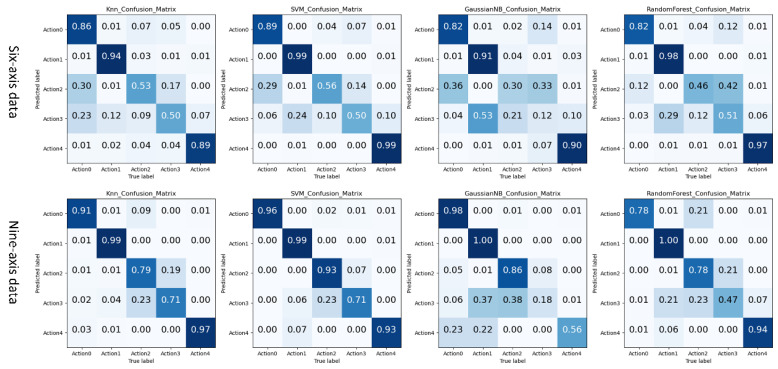
The traditional machine learning model based on artificial features recognizes the result confusion matrix on the professional test set.

Further, we analyzed the recognition results of different classifiers through the confusion matrix graph to judge the performance of the classifiers in different types of actions, as shown in Figure 11.

By analyzing the data in Figure 10 and Figure 11, the following experimental conclusions can be drawn:

i. The machine learning method based on the time domain and frequency domain has a specific classification effect, but it is not ideal.
ii. The average recognition accuracy of 9-axis inertial data is 0.11 higher than that of 6-axis inertial data. More valuable features can be obtained from 9-axis inertial data.
iii. There are differences in the classification performance of the four classifiers on the ping-pong action inertial data set, from large to small as SVM > KNN > RF > GaussianBN.
iv. The classification effect of the five actions shows a specific rule: action 1 and action 4 are easier to identify, action 0 can be determined, and there will be confusion in the recognition results between action 2 and action 3. This classification rule is most evident on the 6-axis test data.
(2) The recognition results of the classifier on the non-professional test set

**Figure 12:**
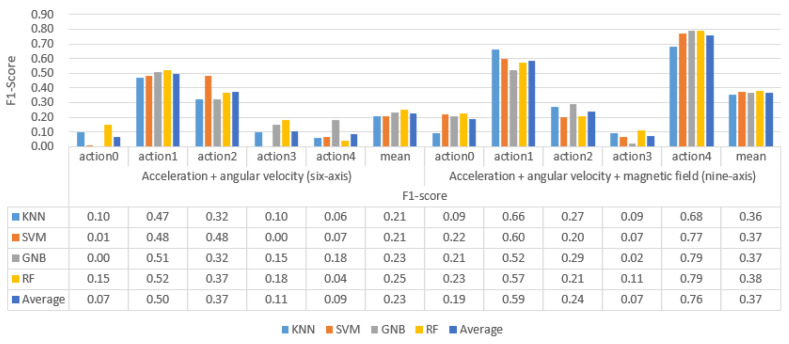
The recognition accuracy of traditional machine learning models based on artificial features on non-professional test sets.

**Figure 13:**
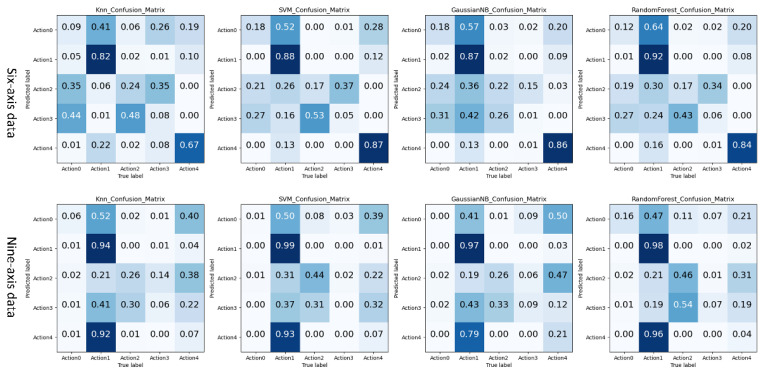
The recognition accuracy of traditional machine learning models based on artificial features on non-professional test sets.

We used the trained classifier to recognize and classify the non-professional test set. The recognition accuracy F1 value and the recognition result confusion matrix are shown in Figure 12 and Figure 13.

By analyzing the data in Figure 12 and Figure 13, the following experimental conclusions can be drawn:

i. Almost all classifiers have lost their classification ability on the 6-axis and 9-axis non-professional test data sets.
ii. The artificially extracted time domain and frequency domain features strongly depend on the data set, and the generalization is poor.

#### 4.2.2. Action Recognition Experiment and Result Analysis Based on Convolutional Neural Network

We used a convolutional neural network to build a classifier. To compare with our proposed multi-dimensional feature fusion convolutional neural network (MDFF-CNN), we built a three-layer convolutional structure of the neural network (CNN) and a layer of the Inception network Convolutional Neural Network (Inception-CNN).

**Figure 14:**
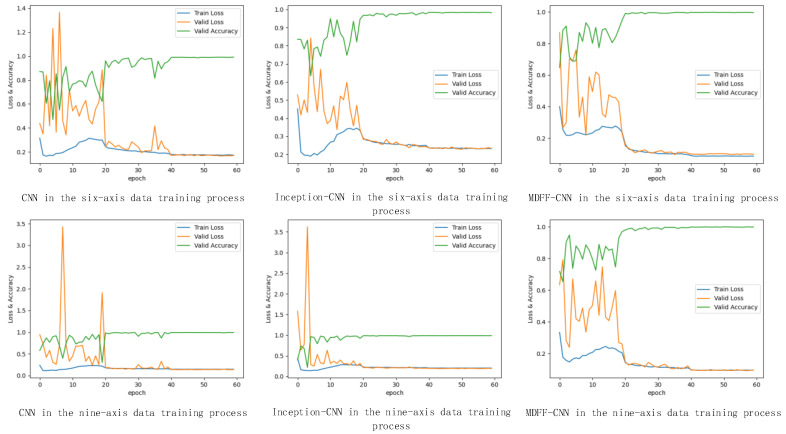
Loss value curve and accuracy curve during convolutional neural network model training.

First, we divided the data into the training set, test set, and validation set at a ratio of 7:3. We used the training set data to train three kinds of neural networks. The process was as follows: After the training data tensor was regularized, it was input into the network in batches, and the data volume of each batch is 64; then, the input data was passed through the network model through the forward algorithm and cross-entropy loss was performed using a Function calculation; in the training phase, the backpropagation algorithm was used, and the Adam optimization function was used to optimize the network parameters. The network weight was not adjusted in the verification or testing phase; the model was trained for 60 generations, and the average loss of the network on the training set and the verification set was calculated once for each generation. We valued and calculated the accuracy of the network on the verification set; the initial learning rate was set to 0.01, and the learning rate was reduced by 0.1 times every 20 generations. The loss function and accuracy curve during the training process are shown in Figure 14. It can be seen from the figure that the prediction accuracy was continuously improving. After 30 generations of training, the network began to converge and finally reached stability.

(1) Recognition results of three types of convolutional neural networks on professional test sets

**Figure 15:**
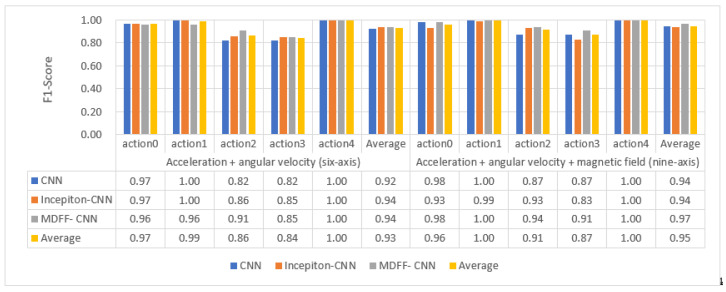
Recognition accuracy rate of convolutional neural network model on professional test set.

**Figure 16:**
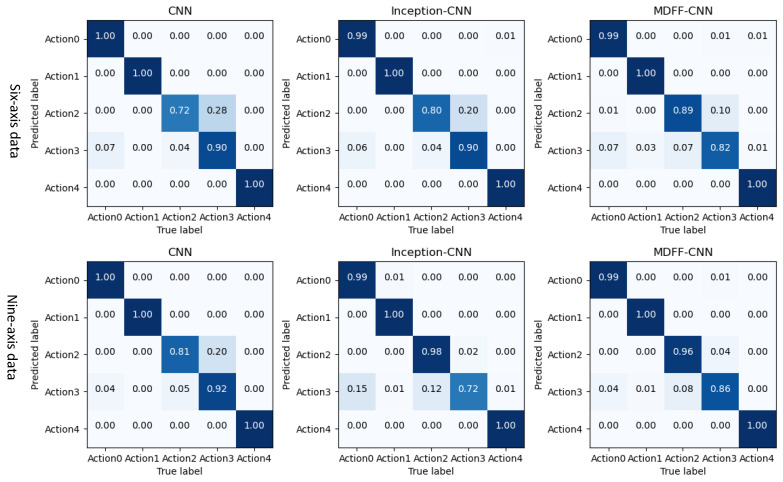
Convolutional neural network model recognizes the result confusion matrix diagram on the professional test set.

The three trained convolutional neural network models were used to identify and classify the professional test set. The recognition accuracy F1 value and the recognition result in the confusion matrix are shown in Figure 15 and Figure 16.

By analyzing the data in Figure 15 and Figure 16, the following experimental conclusions can be drawn:

i. The recognition results of the three convolutional neural network models are all good, the average recognition rate F1 value is above 0.9, and the ideal recognition effect is obtained.
ii. The average recognition rate of 9-axis inertial data is 0.02 higher than that of 6-axis inertial data.
iii. The recognition effect of action 2 and action 3 is lower than the other three actions.
iv. The recognition effect of the three convolutional neural networks on the test set is better than the machine learning classification method based on artificial features.
(2) The recognition results of three convolutional neural networks on the non-professional test set

**Figure 17:**
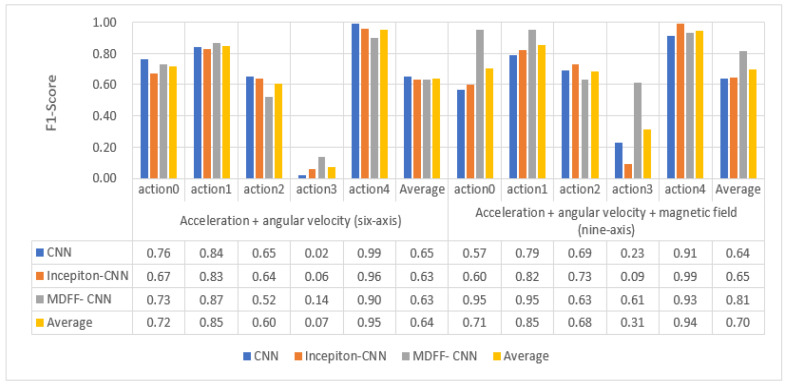
Recognition accuracy rate of convolutional neural network model on non-professional test set.

**Figure 18:**
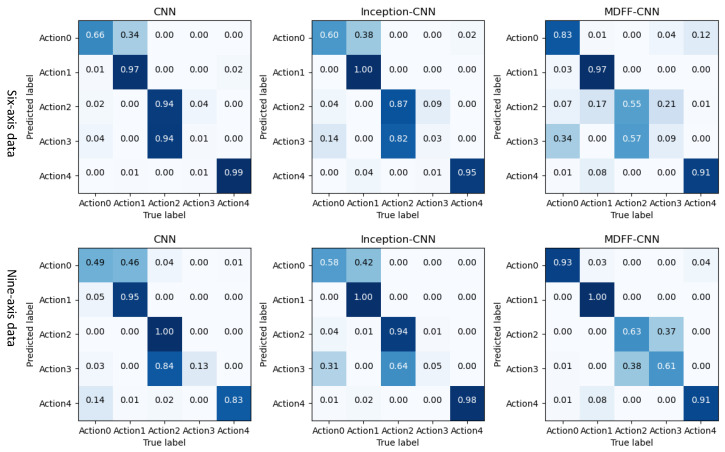
Convolutional neural network model recognizes the result confusion matrix on the non-professional test set.

The three trained convolutional neural network models are used to identify and classify the non-professional test set. The recognition accuracy F1 value and the recognition result in the confusion matrix are shown in Figure 17 and Figure 18.

By analyzing the data in Figure 17 and Figure 18, the following experimental conclusions can be drawn:

① The recognition effect of the three convolutional neural network models on the non-professional test set is lower than the data on the professional test set.

② The recognition effect of the three convolutional neural network models on the non-professional test set is much higher than that of the traditional machine learning model based on artificial features.

③ The average recognition rate of 9-axis inertial data is 0.06 higher than that of 6-axis inertial data.

④ The classification effect of the five actions shows a certain pattern: action 1 and action 4 can be accurately identified, action 0 and action 2 can be identified well, and action 3 is difficult to identify.

⑤ The recognition effect of our proposed MDFF-CNN on the non-professional test set is much higher than the other two convolution models, especially on the 9-axis non-professional test set, where the average recognition rate is 0.17 and 0.16 higher than that of CNN and Inception-CNN, respectively, with better recognition accuracy and generalization performance.

### 4.3. Human Table Tennis Action Assessment Results

**Figure 19:**
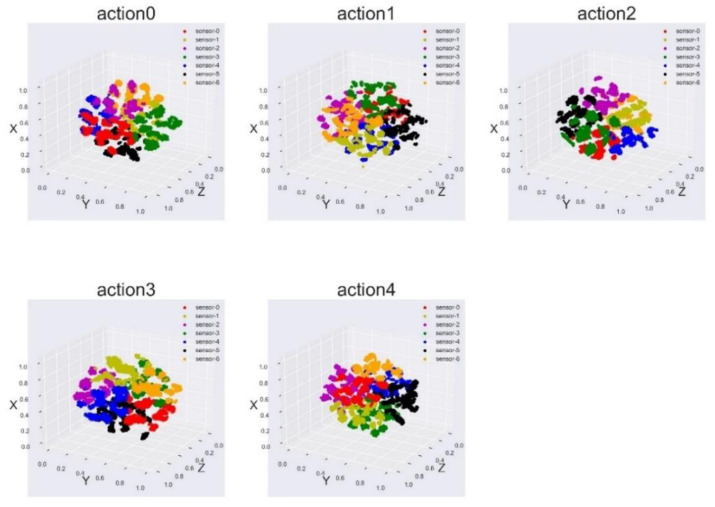
Visualization of local feature vectors of table tennis actions in each category.

We can obtain the local standard features corresponding to different nodes in the five categories of table tennis actions. As shown in Figure 19, we used the table tennis action training data set to cluster the local features of each category of table tennis action. It can be seen from the figure that the characteristic data belonging to the same sensor (local position) in each table tennis action can be gathered together well. The characteristics of sensor data belonging to different locations can be clearly distinguished, indicating that the categories can be well distinguished. The characteristics of sensor data belonging to the same location have a certain degree of dispersion, indicating that there are certain differences within the class, which may be related to the data coming from different subjects.

**Figure 20:**
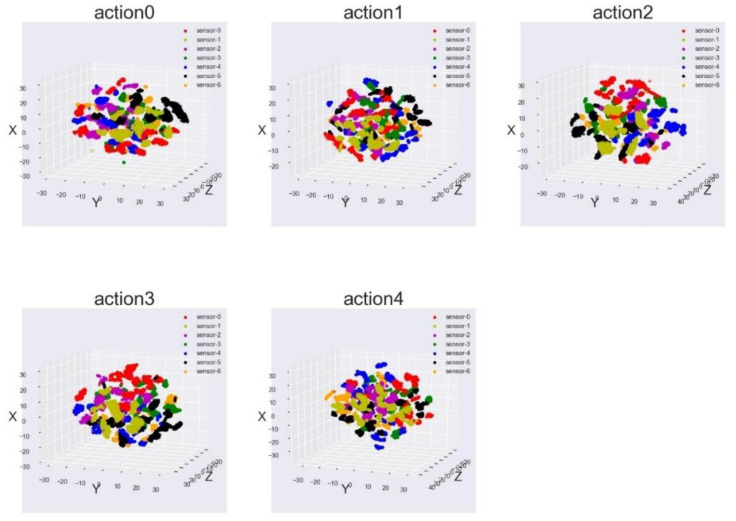
Visualization of local feature vectors of table tennis actions (raw inertial data) for each category.

For comparison, we used the original inertial data of each action, without MDFF-CNN for feature extraction, to build a table tennis standard action model. The original action data frame was separated by the node data, t-SNE dimensionality reduction, and K-means clustering, and then the clustering results are displayed visually, as shown in Figure 20.

It can be seen from Figure 20 that the data of each category cross each other, it is difficult to use K-means to classify the node data, and it is impossible to construct a human table tennis standard motion model from the original inertial data. The 1D and 2D fusion features of the improved Inception acquisition node in the MDFF-CNN network can better represent the local human actions.

Using the K-means clustering algorithm, the nodes with similar characteristics were divided into the same category, and the data represented by the cluster center were used as the standard action feature vector of this type of action at this node (location). The standard motion characteristics obtained using this method are representative, and to a certain extent, can represent the standard table tennis action of the human body.

**Figure 21:**
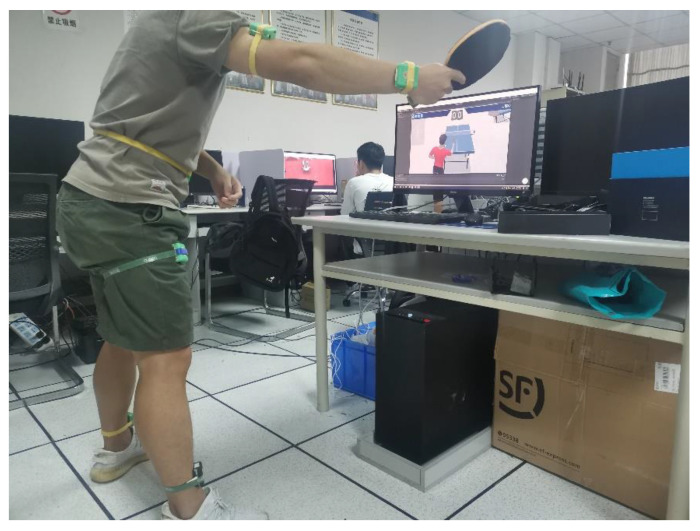
Fast push action training example.

**Figure 22:**
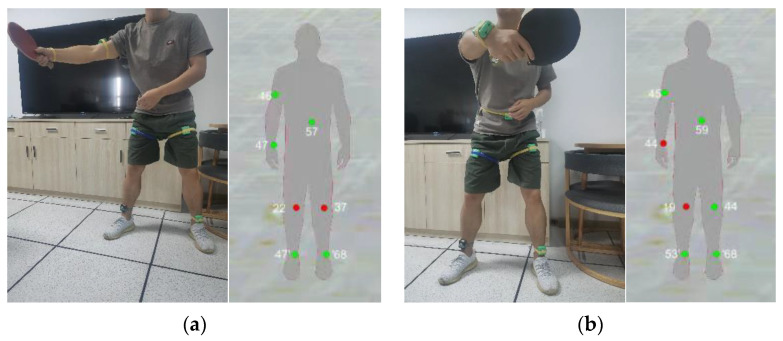
Fast push action of correct and wrong actions and evaluation results. (**a**) is a relatively standardized action. (**b**) subject’s hand action is wrong.

The fly in the ointment is that we believe that there is no quantitative standard for action evaluation. We only reason forward, but there is no reverse verification. Through our method, the final evaluation result is displayed in a visual form. As shown in Figure 21, a subject is performing fast push training. Through the detailed evaluation method of appeal, we displayed the position of the two inertial sensors with the lowest scores or errors to remind users of their deficiencies to achieve the purpose of assisting training. Figure 22 shows correct and incorrect demonstration actions and corresponding evaluations, and the subject is in action at the end of the fast push action in the figure. The fast push action of the subject on the left side of Figure 22 is a relatively standardized action, and the score on the thigh is relatively low. From the right side of Figure 22, it can be seen that the subject’s hand action is wrong. At this time, the node corresponding to the wrong action is turned red.

## 5. Discussion

We propose a new method based on a multi-dimensional feature fusion convolutional neural network and fine-grained evaluation of human table tennis action, which is used to recognize human table tennis action and achieve the purpose of auxiliary training. Among them, the standard model of human body local action is difficult to describe. We propose a new solution: First, use the improved Inception layer in the MDFF-CNN model to have strong representation and generalization capabilities for action data, and the improved cascaded feature map output by the inception of Inception performs a matrix addition operation to obtain the abstract feature of the table tennis action, replaces the original data with the feature matrix, and uses t-SNE to reduce the dimensionality of the node features. Then, use K-Means to cluster the abstract features of all the nodes under each action and use the clustered “cluster center” as the standard model of the local position of the human body. Finally, the cosine similarity algorithm is used to calculate the similarity between the target action and the standard action model, and the similarity is used as the fine-grained evaluation result of the human table tennis action. On this basis, the knowledge of table tennis sports is integrated, and targeted training programs are designed to guide users to improve the standardization of table tennis actions and help users promote health through exercise.

Many issues need to be further improved and studied in depth. We did not analyze the impact of wearing inertial sensors on different positions of the human body on the recognition of ping-pong actions. Moreover, the currently proposed human ping-pong action evaluation method is only forward reasoning, but there is no reverse verification, and the accuracy needs to be further improved. In the future, it will enhance the representativeness of cluster centers and enhance the similarity algorithm from feature representation and clustering. These three aspects will strengthen the accuracy of human ping-pong action assessment.

## Figures and Tables

**Table 1:** Comparison of three action capture technologies.

	Visual Action Recognition	Acoustic Action Recognition	Inertial Action Recognition
Flexibility	General	General	High
Accuracy	Extremely high	Low	High
Sampling frequency	Extremely high	Low	High
Movable range	Small	General	Big
Computational efficiency	General	General	High
Multi-target capture	General	General	High
Cost	High	Low	Low
Calibration time	Long	General	Short
Environmental constraints	Strong light, Occlusion	Temperature, air	Magnetic interference

**Table 2:** Personal attributes of professional table tennis players.

Serial Number	Gender	Age	Height (cm)	Weight (kg)
Person 0	Man	24	175	70
Person 1	Woman	24	158	47
Person 2	Man	21	172	72
Person 3	Woman	22	168	53
Person 4	Woman	22	163	65
Person 5	Woman	21	160	50
Person 6	Woman	22	155	60
Person 7	Man	27	165	68
Person 8	Man	27	175	79
Person 9	Man	23	176	72
Person 10	Man	22	185	75
Person 11	Woman	28	163	55

**Table 3:** Collecting data table of five table tennis hitting actions.

Action Name	Number of Actions	Number of Data Frames
Forehand Stroke	200	12,549
Fast push	200	12,619
Speedo	200	12,449
Loop	200	11,899
Backhand Push	200	13,287
Sum	1000	62,803

**Table 4:** Experimental data set.

Action	Training Sets	Validation Sets	Major-Test Sets	Amateur-Test Sets
Action 0	1411	389	200	180
Action 1	1402	398	200	180
Action 2	1384	416	200	180
Action 3	1398	402	200	180
Action 4	1405	395	200	180
Sum	7000	2000	1000	900
